# GRHL2-HER3 and E-cadherin mediate EGFR-bypass drug resistance in lung cancer cells

**DOI:** 10.3389/fcell.2024.1511190

**Published:** 2025-01-17

**Authors:** Fumiya Ito, Wakiko Iwata, Yoshihiro Adachi, Hiromi Sesaki, Miho Iijima

**Affiliations:** Department of Cell Biology, Johns Hopkins University School of Medicine, Baltimore, MD, United States

**Keywords:** EGFR, lung cancer, drug resistance, GRHL2, HER3, cadherin

## Abstract

Epidermal growth factor receptor (EGFR) is a major oncogenic protein, and thus EGFR-targeting therapies are widely used in patients with various types of cancer, including lung cancer. However, resistance to EGFR inhibitors, such as erlotinib, presents a significant challenge in treating lung cancer. In this study, we established an EGFR-independent, erlotinib-resistant (ER) phenotype in lung cancer A549 cells by exposing them to erlotinib for an extended period. The resulting ER cells exhibited a dramatic increase in erlotinib resistance, a decreased EGFR protein level, and enhanced tumor growth, suggesting a robust mechanism bypassing EGFR inhibition. RNA sequencing identified the transcription factor GRHL2 as a critical player in this resistance. GRHL2 was upregulated in ER cells, and its knockdown and knockout significantly reduced erlotinib resistance. Further analysis revealed that GRHL2 upregulates the receptor tyrosine kinase HER3, and that HER3 knockdown similarly decreases the IC_50_ for erlotinib. Additionally, ER cells showed increased cell-cell adhesion, linked to upregulated E-cadherin. E-cadherin was found to be vital for erlotinib resistance, largely independent of GRHL2, highlighting multiple parallel pathways sustaining resistance. These findings provide a novel mechanism of drug resistance and suggest that combination therapies targeting both GRHL2-HER3 and E-cadherin-mediated pathways may be necessary to overcome erlotinib resistance in lung cancer.

## Introduction

Lung cancer is the leading cause of cancer-related mortality worldwide and divided into two major types: small cell lung cancer (SCLC) and non-small cell lung cancer (NSCLC) (Herbst et al., 2018; Leiter et al., 2023; Liu et al., 2023). NSCLC is the most common type, accounting for approximately 85%–90% of all lung cancer cases (Herbst et al., 2018; Leiter et al., 2023; Liu et al., 2023). The epidermal growth factor receptor (EGFR), which facilitates cell growth and proliferation, is a primary oncogenic driver of NSCLC (Herbst et al., 2018; Gong et al., 2020; Leiter et al., 2023; Liu et al., 2023). Genetic mutations in the EGFR gene occur in approximately 20%–50% of patients with NSCLC, depending on their ethnic background (Rosell et al., 2009; Pao and Chmielecki, 2010; Cancer Genome Atlas Research, 2014; Shi et al., 2014). As a result, cancer therapies that target EGFR, using drugs that directly bind to the receptor, have been extensively used in clinical treatments for EGFR-positive NSCLC, and their strong efficacy has been well-established (Herbst et al., 2018; Pan et al., 2023). However, cancer cells can develop drug resistance during long-term treatments, which diminishes the effectiveness of EGFR inhibitors (Rotow and Bivona, 2017; Herbst et al., 2018; Fu et al., 2022). Drug resistance poses significant challenges in cancer therapy, with approximately 80%–90% of cancer-related mortality attributed to it (Borst, 2012; Housman et al., 2014; Alfarouk et al., 2015; Herbst et al., 2018; Fu et al., 2022; Saini et al., 2023). Overcoming resistance to EGFR inhibition and improving anticancer outcomes require an urgent and deeper understanding of cellular responses to EGFR inhibitors.

Previous studies have demonstrated that, during treatment with EGFR inhibitors, cancer cells can acquire somatic mutations in the EGFR gene, significantly reducing the drugs’ affinity for EGFR (Rosell et al., 2009; Yu et al., 2014; Sattler et al., 2023; Tomuleasa et al., 2024). For example, the T790M point mutation, which substitutes threonine at position 790 with methionine, has been reported in patients who develop resistance to FDA-approved EGFR inhibitors, such as the first-generation drug erlotinib (Pao et al., 2005; Tang et al., 2013; Karachaliou et al., 2019). This mutation enhances the interaction between the kinase domain of EGFR and ATP. To overcome this on-target, EGFR-mediated drug resistance, subsequent generations of EGFR inhibitors have been developed. Among these, osimertinib, a third-generation FDA-approved EGFR inhibitor, has been shown to inhibit both the initial oncogenic EGFR mutation and the resistance-associated T790M mutation (Soria et al., 2018; Passaro et al., 2021). However, even with the highly effective third generation of EGFR inhibitors, their anticancer effects can gradually diminish, and a population of cancer cells with additional resistance often emerges, presenting critical challenges that need to be addressed (Leonetti et al., 2019; Ramalingam et al., 2020; Cooper et al., 2022; Fu et al., 2022; Gomatou et al., 2023). The molecular basis of this secondary resistance could involve further mutations in the EGFR gene, such as substituting the cysteine residue at position 797, which lowers osimertinib’s affinity for EGFR by disrupting its covalent binding (Leonetti et al., 2019; Tomuleasa et al., 2024). Additionally, amplification and upregulation of EGFR can contribute to resistance against EGFR inhibitors (Leonetti et al., 2019; Tomuleasa et al., 2024).

In addition to the on-target drug resistance mechanisms that directly alter EGFR, other mechanisms that do not involve mutations or amplification of the EGFR gene have been increasingly recognized (Chong and Janne, 2013; Sever and Brugge, 2015; Rotow and Bivona, 2017; Leonetti et al., 2019; Passaro et al., 2021; Cooper et al., 2022; Tomuleasa et al., 2024). Understanding these EGFR-independent or -bypass mechanisms has been more challenging than on-target mechanisms, as it requires identifying genetic changes beyond EGFR and potential non-genomic alterations (Leonetti et al., 2019; Tomuleasa et al., 2024). Despite these challenges, studies have begun to unravel these mechanisms, revealing that EGFR-bypass adaptive mechanisms involve changes in other receptor tyrosine kinases, such as the amplification of MET (mesenchymal-epithelial transition factor receptor) and the rearrangement of RET (rearranged during transfection) (Chong and Janne, 2013; Rotow and Bivona, 2017; Leonetti et al., 2019; Passaro et al., 2021; Cooper et al., 2022; Tomuleasa et al., 2024). These receptor kinases can independently activate oncogenic signaling cascades, bypassing the need for EGFR and allowing disease progression even when EGFR is blocked by its inhibitors (Chong and Janne, 2013; Sever and Brugge, 2015; Rotow and Bivona, 2017; Leonetti et al., 2019; Passaro et al., 2021; Cooper et al., 2022; Tomuleasa et al., 2024). Beyond these alterations in receptor tyrosine kinases, the activation of downstream components in signal transduction pathways, such as AKT and ERK, has also been proposed as off-target resistance mechanisms (Chong and Janne, 2013; Sever and Brugge, 2015; Rotow and Bivona, 2017; Leonetti et al., 2019; Passaro et al., 2021; Cooper et al., 2022; Tomuleasa et al., 2024). However, the crucial bypass mechanisms identified so far likely represent just the tip of the iceberg. Fully uncovering the landscape of off-target mechanisms is essential to developing more effective therapeutic strategies for cancer treatment. Towards this goal, in this study, we established resistance to EGFR inhibition in NSCLC cells and identified novel parallel mechanisms by which the transcription factor, grainyhead-like 2 (GRHL2), and E-cadherin drive drug resistance.

## Materials and methods

### Cells

A549, Calu-1, SK-LU-1, and HEK293T cells were obtained from ATCC. All cell lines were cultured under standard conditions (37°C in a humidified atmosphere with 5% CO_2_) in DMEM containing 10% FBS (Nguyen et al., 2014; Nguyen et al., 2015; Yang et al., 2017; Igarashi et al., 2018; Kato et al., 2020). To establish erlotinib-resistant cells (ER cells), cells were cultured in the presence of 15–30 μM erlotinib (HY-50896, MedChemExpress) for 6 months. The culture medium containing erlotinib was replaced every 3–4 days. After 6 months, cells were frozen in a medium containing 90% FBS and 10% DMSO and stored in liquid nitrogen. ER cells were cultured in DMEM containing 10% FBS and 10 µM erlotinib at 37°C with 5% CO_2_. Afuresertib (S7521) and Ipatasertib (S2808) were purchased from Selleckchem.

### Spheroid growth and invasion

0.5–1.0 × 10^4^ cells were resuspended in 50 µL of Geltrex (A1413202, Thermo Fisher), placed in 8-well chambered coverglasses, and incubated at 37°C for 30 min to allow the Geltrex to solidify. Subsequently, 250 µL of DMEM containing 10% FBS was added. The cells were allowed to form spheroids for 7 days in a cell culture incubator at 37°C with 5% CO_2_. After 7 days, the spheroids were collected using Corning Cell Recovery Solution (CLS354253, Millipore Sigma) and then resuspended at a concentration of 1 spheroid per 1 µL of Geltrex in 24-well plates coated with Sigmacote (SL2, Millipore Sigma). The plates were incubated at 37°C for 30 min to allow the Geltrex to solidify, after which 500 µL of DMEM containing 10% FBS was added. The culture medium was changed on day 3 or 4. Images of cells and spheroids were captured using phase-contrast microscopy (AXIO Observer Z1, Zeiss). The total spheroid area was calculated using NIH Fiji software.

### Western blotting

Cells were lysed in lysis buffer (1% NP40, 10% glycerol, 50 mM NaCl, 20 mM Tris-HCl [pH 7.4]) supplemented with cOmplete, Mini, EDTA-free Protease Inhibitor Cocktail (11836170001, Sigma) on ice (Nguyen et al., 2014; Nguyen et al., 2015; Yang et al., 2017; Igarashi et al., 2018; Senoo et al., 2019; Kato et al., 2020; Senoo et al., 2021). The lysates were centrifuged at 16,000 g for 10 min at 4°C, and the supernatants were collected. Proteins were separated by SDS-PAGE and transferred onto Immobilon-FL Transfer Membranes (Millipore). The membranes were blocked in PBS-T (PBS containing 0.05% Tween 20) supplemented with 3% BSA at room temperature for 30 min and then incubated with primary antibodies in PBS-T supplemented with 3% BSA at 4°C overnight. The antibodies used are GRHL2 (ab271023, Abcam), HER3 (12,708, Cell Signaling), E-cadherin (14,472, Cell Signaling), β-catenin (ab32572, Abcam), EGFR (4267, Cell Signaling), AKT (9272, Cell Signaling), phospho-AKT at S473 (4060, Cell Signaling), p38 (8690, Cell Signaling), phospho-p38 (4511, Cell Signaling), ERK1/2 (9107, Cell Signaling), phospho-ERK1/2 (4370, Cell Signaling), GAPDH (MA5-15738, Invitrogen), PARP (9542, Cell Signaling), Caspase-3 (9665, Cell Signaling), and LC3 (PM036, MBL). The membranes were washed three times in PBS-T, followed by incubation with appropriate fluorescently labeled secondary antibodies at room temperature for 1 h. After washing the membranes three times in PBS-T, fluorescence signals were detected using a Typhoon biomolecular imager (Amersham). Images were analyzed using NIH Fiji software.

### Plasmids

The following sequences were cloned into pLKO.1 puro (8453, Addgene) to generate shRNA plasmids (Adachi et al., 2020; Murata et al., 2024). Scramble: CCT​AAG​GTT​AAG​TCG​CCC​TCG​CTC​GAG​CGA​GGG​CGA​CTT​AAC​CTT​AGG; LEF1: CCA​CAC​TGA​CAG​TGA​CCT​AAT​CTC​GAG​ATT​AGG​TCA​CTG​TCA​GTG​TGG, CCA​TCA​GAT​GTC​AAC​TCC​AAA​CTC​GAG​TTT​GGA​GTT​GAC​ATC​TGA​TGG, GCA​CGG​AAA​GAA​AGA​CAG​CTA​CTC​GAG​TAG​CTG​TCT​TTC​TTT​CCG​TGC; GRHL2: GCC​GAT​TAC​AAG​GAG​AGC​TTT​CTC​GAG​AAA​GCT​CTC​CTT​GTA​ATC​GGC, CCT​TCA​AAG​CAG​ATG​AAA​GAA​CTC​GAG​TTC​TTT​CAT​CTG​CTT​TGA​AGG, GCT​GAA​GAT​TTC​ACA​CCA​GTT​CTC​GAG​AAC​TGG​TGT​GAA​ATC​TTC​AGC; EVX1: CGC​CTT​CTA​CAC​TTA​CAT​GAT​CTC​GAG​ATC​ATG​TAA​GTG​TAG​AAG​GCG, GTC​GGA​TTT​CTA​TGA​AGA​AAT​CTC​GAG​ATT​TCT​TCA​TAG​AAA​TCC​GAC, CCG​CCC​TAA​ACC​TGC​CGG​AAA​CTC​GAG​TTT​CCG​GCA​GGT​TTA​GGG​CGG; ZFP57: AGC​AAG​TCT​TTC​AGC​TCA​TTT​CTC​GAG​AAA​TGA​GCT​GAA​AGA​CTT​GCT, TGT​TAT​GTC​GGA​AAC​CTT​TAA​CTC​GAG​TTA​AAG​GTT​TCC​GAC​ATA​ACA, AGG​TCC​CAG​GAA​CCC​ATA​TTT​CTC​GAG​AAA​TAT​GGG​TTC​CTG​GGA​CCT; MEF2C: GCC​TAG​AAT​TTG​ATA​CGC​TTT​CTC​GAG​AAA​GCG​TAT​CAA​ATT​CTA​GGC, CGT​GGA​GAC​GTT​GAG​AAA​GAA​CTC​GAG​TTC​TTT​CTC​AAC​GTC​TCC​ACG, CCC​AAT​GAA​TTT​AGG​AAT​GAA​CTC​GAG​TTC​ATT​CCT​AAA​TTC​ATT​GGG; HER3: AGG​TTA​GGA​GTA​GAT​ATT​GAC​TCG​AGT​CAA​TAT​CTA​CTC​CTA​ACC​T, AAT​TCT​CTA​CTC​TAC​CAT​TGC​TCG​AGC​AAT​GGT​AGA​GTA​GAG​AATT,​ TAT​ATG​AAT​CGG​CAA​CGA​GAT​CTC​GAG​ATC​TCG​TTG​CCG​ATT​CAT​ATA; E-cadherin (CDH1): CCA​AGC​AGA​ATT​GCT​CAC​ATT​CTC​GAG​AAT​GTG​AGC​AAT​TCT​GCT​TGG, CGA​TTC​AAA​GTG​GGC​ACA​GAT​CTC​GAG​ATC​TGT​GCC​CAC​TTT​GAA​TCG, CCA​ACC​CAA​GAA​TCT​ATC​ATT​CTC​GAG​AAT​GAT​AGA​TTC​TTG​GGT​TGG.

To express GFP, pLentiCMV-GFP-Puro was generated by removing the Met gene from pLentiCMV-MetGFP-Puro (Addgene, 37,560). To express HER3, pLentiCMV-HER3-Puro was generated by replacing GFP with HER3, which was cloned from cDNA of A549 cells, in pLentiCMV-GFP-Puro. To express E-cadherin, pHAGE-CDH1 was used (Addgene, 116722).

### Lentivirus production

HEK293T cells were seeded at 5 × 10^5^ cells per well in a 6-well dish and cultured for 24 h (Adachi et al., 2020; Murata et al., 2024). pLX313 carrying firefly luciferase (118,017, Addgene), pLKO.1 puro carrying shRNAs, pLentiCMV-GFP-Puro, pLentiCMV-HER3-Puro, or pHAGE-CDH1was co-transfected into HEK293T cells along with pCMV-VSVG and pHR-CMV8.2ΔR using polyethylenimine (23966-1, Polysciences, Inc.) (Adachi et al., 2020; Murata et al., 2024). The culture medium was replaced 3 h after transfection. Culture media containing virus particles were collected 48 h after transfection. The virus was aliquoted and stored at −80°C. For lentiviral transduction, cells were seeded at 5 × 10^5^ cells/well in a 6-well plate and cultured for 24 h. Cells were then incubated with lentivirus in DMEM containing 10% FBS and 8 μg/mL polybrene for 24 h.

### Tumorigenicity assay

All work involving animals was conducted according to the guidelines established by the Johns Hopkins University Committee on Animal Care and Use. Nude mice (strain number: 002019) were purchased from The Jackson Laboratory. The tumorigenicity assay was conducted as described previously (Pirazzoli and Politi, 2014; Ota et al., 2016; Du et al., 2017). Cells were infected with lentiviruses expressing luciferase and selected for 7 days with hygromycin (250 μg/mL). A total of 1 × 10^6^ cells were resuspended in 100 μL of PBS and injected subcutaneously into the dorsal skin of nude mice. For erlotinib treatment, mice were subjected to intraperitoneal injection at 25 mg/kg/day every Monday through Friday for 4 weeks (Politi et al., 2010; Wu et al., 2013). To monitor tumor development, mice were injected with 150 μL D-luciferin (30 mg/mL) for 10 min and analyzed using the *In Vivo* Imaging System (IVIS, Xenogen). Total photon counts were quantified using Living Image software 4.7.4. Tumors were dissected at 4 weeks, and their weight was measured.

### DNA sequencing of EGFR

The region spanning exons 18–21 of the EGFR gene was PCR amplified from the cDNA of ER cells using the primers TCG​GCC​TCT​TCA​TGC​GAA​GGC and CAA​TGC​CAT​CCA​CTT​GAT​AGG​CAC. The PCR products were then analyzed by DNA sequencing.

### RNA sequencing and data analysis

Total RNA isolation, RNA library preparation, and transcriptome sequencing were conducted by Novogene (Sacramento, CA). Genes with an adjusted p-value <0.01 and a log2 fold change >8 were considered differentially expressed genes (DEGs). Transcription factors were selected based on the RNA-seq analysis data provided by Novogene.

### Cell aggregation assay

To prepare a low-adhesion culture plate, 24-well cell culture plates were treated with 500 μL Sigmacoat (SL2-25 mL, Sigma-Aldrich) according to the manufacturer’s protocol. Before using the coated plate, wells were washed twice with 500 μL PBS. A total of 5 × 10^4^ cells in 0.5 mL were added to the 24-well coated plates and incubated for 24 h on a rotary shaker at 125 rpm at 37°C with 8% CO_2_. Images were obtained using EVOS Cell Imaging Systems (Thermo Fisher) and a 10x/0.30 NA EVOS objective lens (AMEP4981, Invitrogen). Images were analyzed using NIH Fiji software.

### esiRNA knockdown

ER cells were plated at 1 × 10⁵ cells per well in a 12-well plate and cultured for 24 h esiRNAs targeting E-cadherin (EHU090371, Sigma) or a Renilla luciferase-targeting negative control (EHURLUC, Sigma) were transfected into the ER cells using Lipofectamine RNAiMAX (Invitrogen) for 3 days. The cells were then used for Western blotting and the cell aggregation assay.

### Gene knockout

Gene knockout of GRHL2 and E-cadherin (CDH1) was performed using the GeneArt CRISPR Nuclease (OFP reporter) vector kit (A21174; Thermo Fisher) according to the manufacturer’s instructions (Adachi et al., 2020; Murata et al., 2024). The following target gRNA sequences were used: GRHL2: CCG​AAG​AGC​CTA​CAC​CAG​TG, E-cadherin: AAG​ATT​GCA​CCG​GTC​GAC​AA.

### qPCR

Cells were plated in 24-well plates, and total RNA was extracted using the GeneJET RNA Purification Kit (K0732; Thermo Fisher). The RNA was reverse-transcribed into cDNA using the ReadyScript cDNA Synthesis Mix (RDRT, Sigma-Aldrich). Quantitative PCR (qPCR) was performed on a QuantStudio 3 Real-Time PCR System (Applied Biosystems) with PowerUp SYBR Green Master Mix (A25741, Thermo Fisher Scientific). GAPDH served as the reference gene. The following primers were used: HER3: CAA​CTC​TCA​GGC​AGT​GTG​TCC and CCA​TCA​CCA​CCT​CAC​ACC​TC, GAPDH: AAG​GTC​ATC​CCT​GAG​CTG​AA and CTG​CTT​CAC​CAC​CTT​CTT​GA.

### Statistical analyses

Statistical analysis was performed using GraphPad Prism. Specific statistical tests and significance thresholds are detailed in the figure legends.

## Results

### EGFR-bypass erlotinib resistance in NSCLC cells

**FIGURE 1:**
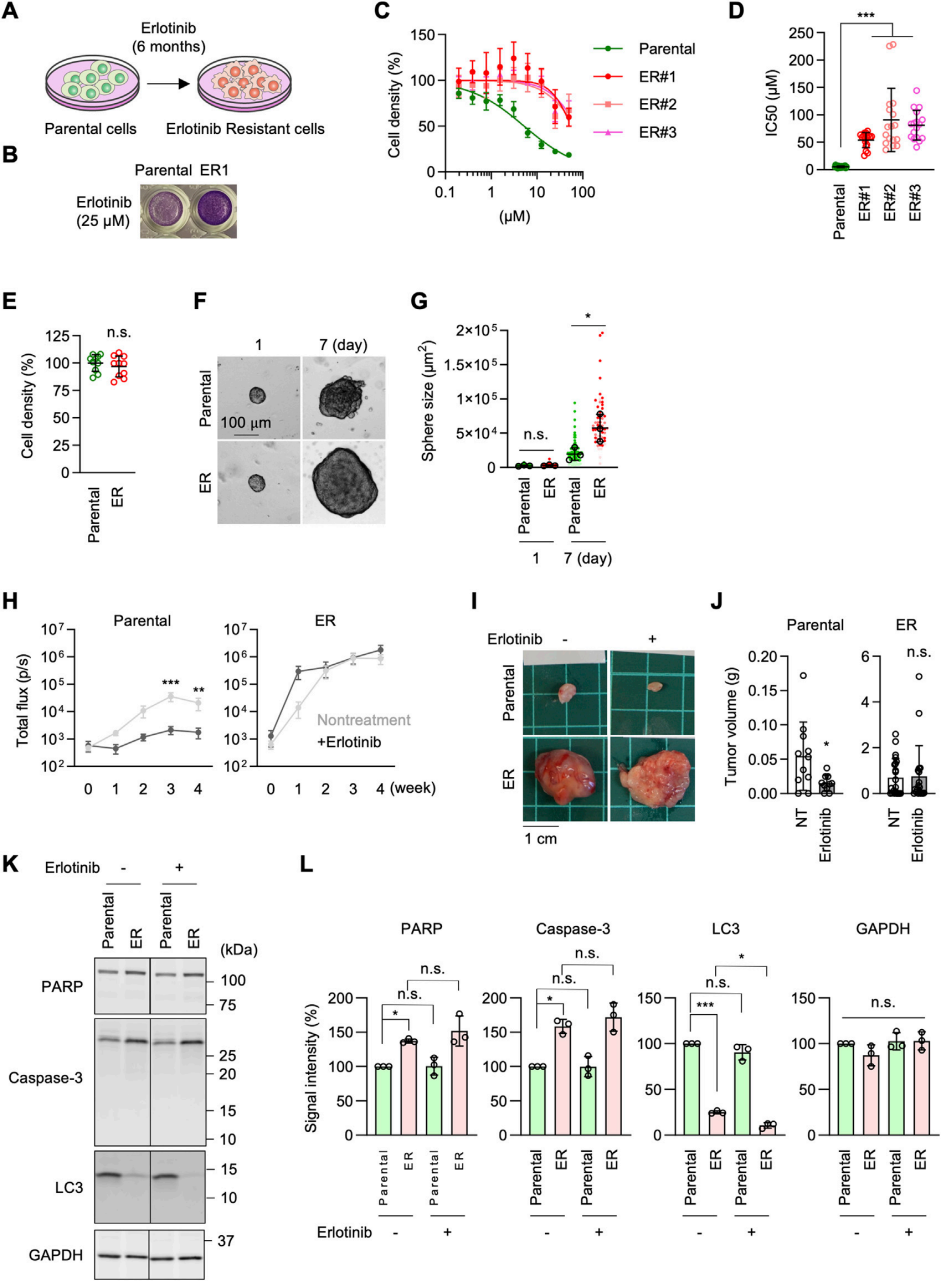
Generation of erlotinib-resistant A549 cells. **(A)** Parental A549 cells were cultured in 10–30 µM erlotinib to select erlotinib-resistant cells (ER cells). **(B)** Parental and ER cells were cultured in 25 µM erlotinib for 72 h and stained with crystal violet. **(C)** Parental and three independent ER cell lines were cultured in the presence of 0–50 µM erlotinib for 72 h. Cell density was determined by crystal violet staining. Relative cell density is quantified. Values are mean ± SD (n = 18). **(D)** IC_50_ of Parental and ER cells is presented. Values are mean ± SD (n = 18 for Parental cells, n = 17 for ER cells). **(E)** Cell proliferation was assessed without erlotinib using crystal violet staining. Values are mean ± SD (n = 10). **(F)** Parental and ER spheroids were placed in Geltrex and monitored for 7 days **(G)** Quantification of the total area. Mean ± SD (n = 3). **(H)** Luciferase-expressing Parental and ER cells were subcutaneously injected into nude mice. For erlotinib treatment, mice were subjected to intraperitoneal injection at 25 mg/kg/day every Monday through Friday for 4 weeks. Bioluminescence was measured and quantified at the indicated time points. Mean ± SEM (n = 9 nontreated and 10 treated Parental cells and 10 nontreated and 9 treated ER cells). **(I)** Representative images of tumors generated from Parental and ER cells at 4 weeks **(J)** Tumor weight was measured. Mean ± SD (n = 11 nontreated and 10 treated Parental cells, 22 nontreated and 19 treated ER cells). **(K)** Western blotting of Parental and ER cells using antibodies to caspase-3, PARP, LC3, and GAPDH. (**L**) Quantification of band intensity. Mean ± SD (n = 3). One-way ANOVA with post hoc Tukey in (**D, G, L**) and Šídák **(H)**, and two-tailed Student’s t-test in **(J)**: *p < 0.05, **p < 0.01, ***p < 0.001.

To establish EGFR inhibition resistance in NSCLC cells, we cultured A549 NSCLC cells in the presence of the EGFR inhibitor erlotinib at 15–30 µM for 6 months (Figure 1A). This range of concentrations was chosen as sublethal doses based on our preliminary experiments, in which the proliferation of A549 cells was tested in the presence of 5–30 µM erlotinib. The prolonged erlotinib treatment was performed in three separate plates of cell cultures to obtain multiple independent resistant cell lines. After 6 months, A549 erlotinib-resistant cells (ER cells) from each culture showed resistance to erlotinib, with a dramatically increased half-maximal inhibitory concentration (IC_50_) for cell proliferation, approximately 10-fold higher compared to the original A549 cells (Parental cells) (Figures 1B–D). In the absence of erlotinib, ER cells proliferated at a similar rate to Parental cells (Figure 1E). We also treated two other lung cancer cell lines, Calu-1 and SK-LU-1, with erlotinib for 6 months. We found that, similar to A549 cells, SK-LU-1 cells acquired resistance to erlotinib, whereas Calu-1 cells did not (Supplementary Figure S1). In this study, we focused on characterizing A549 ER cells.

To characterize ER cells under more physiological conditions, we cultured Parental and ER cells in Geltrex, an extracellular matrix composed of laminin, collagen IV, entactin, and heparin sulfate proteoglycans (El Harane et al., 2023). We found that ER spheroids grew much faster than Parental spheroids in this 3D culture condition (Figures 1F,G). Since Geltrex contains a mixture of laminin, collagen IV, entactin, and heparan sulfate proteoglycans, these ECM components may preferentially enhance the proliferation of ER cells. Alternatively, cells in Geltrex may experience limited oxygen availability, with resistant cells proliferating better under modestly hypoxic conditions. Consistent with this finding, when Parental and ER cells expressing luciferase were subcutaneously injected into nude mice, bioluminescence imaging showed that ER cells generated much larger tumors than Parental cells (Figure 1H). Similarly, ER cell-derived tumors were significantly heavier than those derived from Parental cells at 4 weeks after injection (Figures 1I, J). Notably, the development of tumors derived from Parental cells was significantly reduced when mice were treated with erlotinib, as indicated by bioluminescence imaging (Figure 1H) and tumor weight (Figures 1I, J). Conversely, erlotinib treatment did not affect the growth of tumors derived from ER cells (Figures 1H–J). These data demonstrate that ER tumors resist EGFR inhibition *in vivo*, which is consistent with the *in vitro* data.

To determine whether ER cells exhibit altered cell survival in response to erlotinib, we compared the effects of erlotinib on apoptosis and autophagy. Parental and ER cells were treated with 5 µM erlotinib for 24 h and analyzed by Western blotting using antibodies against apoptotic markers, including caspase-3 activation and PARP cleavage, as well as LC3 molecular species. We observed increased levels of caspase-3 but no evidence of its proteolytic activation or cleavage of its substrate, PARP, in either parental or ER cells treated with erlotinib (Figures 1K, L). Additionally, although LC3-I levels were decreased in ER cells compared to Parental cells, no conversion to active LC3-II was detected in either Parental or ER cells, regardless of erlotinib treatment (Figures 1K, L). These findings suggest that erlotinib does not induce apoptotic or autophagic cell death in either Parental or ER cells.

**FIGURE 2:**
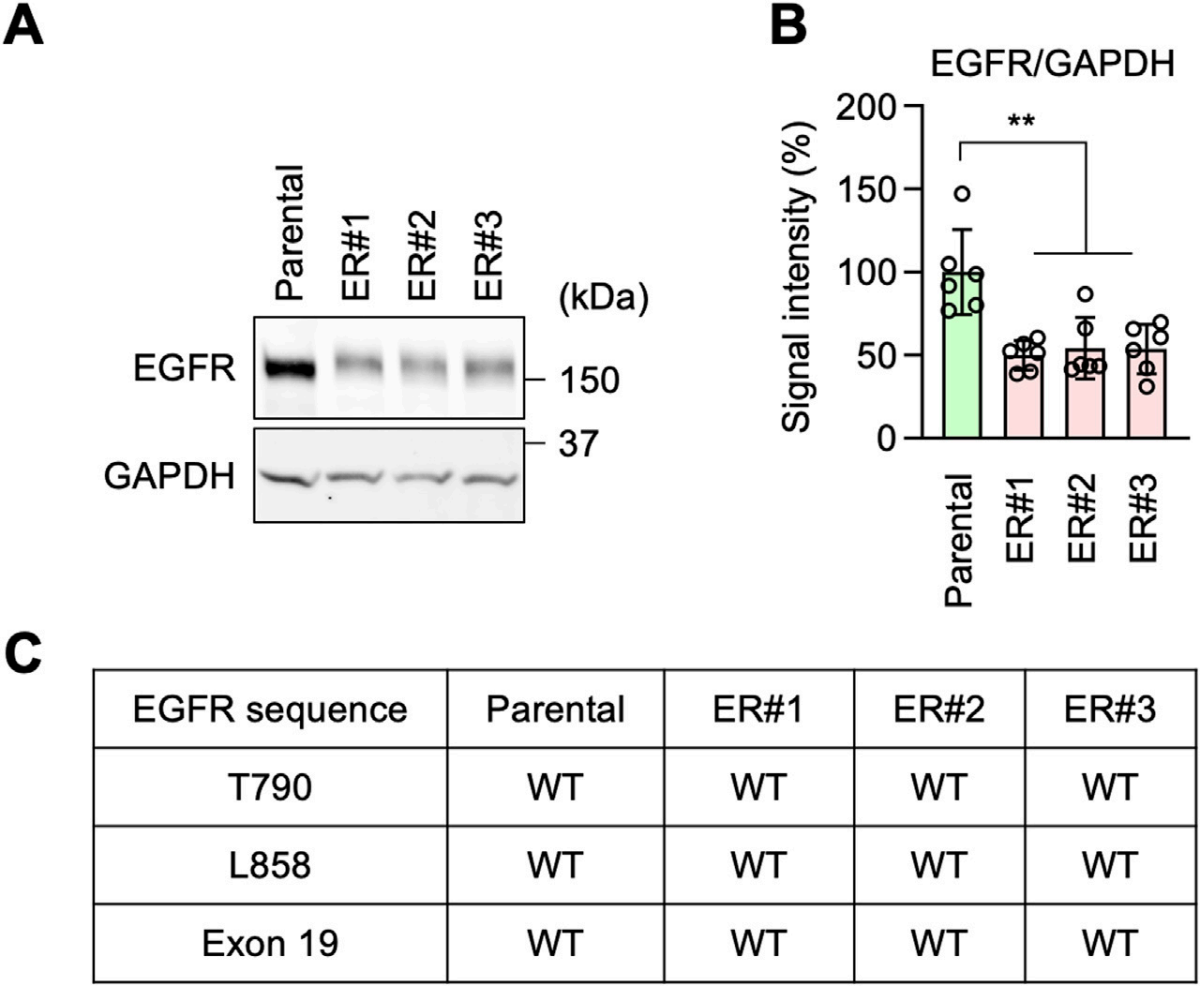
EGFR-bypass resistance in ER cells. **(A)** Western blotting of Parental and ER cells using antibodies to EGFR and GAPDH. **(B)** Quantification of band intensity. Mean ± SD (n = 6). **(C)** The EGFR gene was cloned from the indicated cells and analyzed for mutations and deletions using DNA sequencing. One-way ANOVA with post hoc Tukey in **(B)**: **p < 0.01.

It has been reported that resistance to EGFR inhibition can be acquired through increased expression levels of EGFR, mutations in the EGFR gene, or partial deletions of the gene (Rosell et al., 2009; Yu et al., 2014; Vasan et al., 2019; Passaro et al., 2021; Cooper et al., 2022; Sattler et al., 2023; Tomuleasa et al., 2024). Accordingly, to investigate the mechanism underlying erlotinib resistance in ER cells, we tested the expression levels of EGFR by Western blotting. Surprisingly, we found that EGFR protein levels were decreased, rather than increased, in ER cells compared to Parental cells (Figures 2A, B). Therefore, the expression level of EGFR does not account for the resistance. We also analyzed the DNA sequence of the EGFR gene in ER cells. We found no resistance-associated mutations, such as T790M or L858R, nor any deletion of exon 19 in ER cells (Figure 2C). These data suggest that ER cells have established an EGFR-bypass drug resistance.

### GRHL2 is critical for EGFR-bypass resistance

**FIGURE 3:**
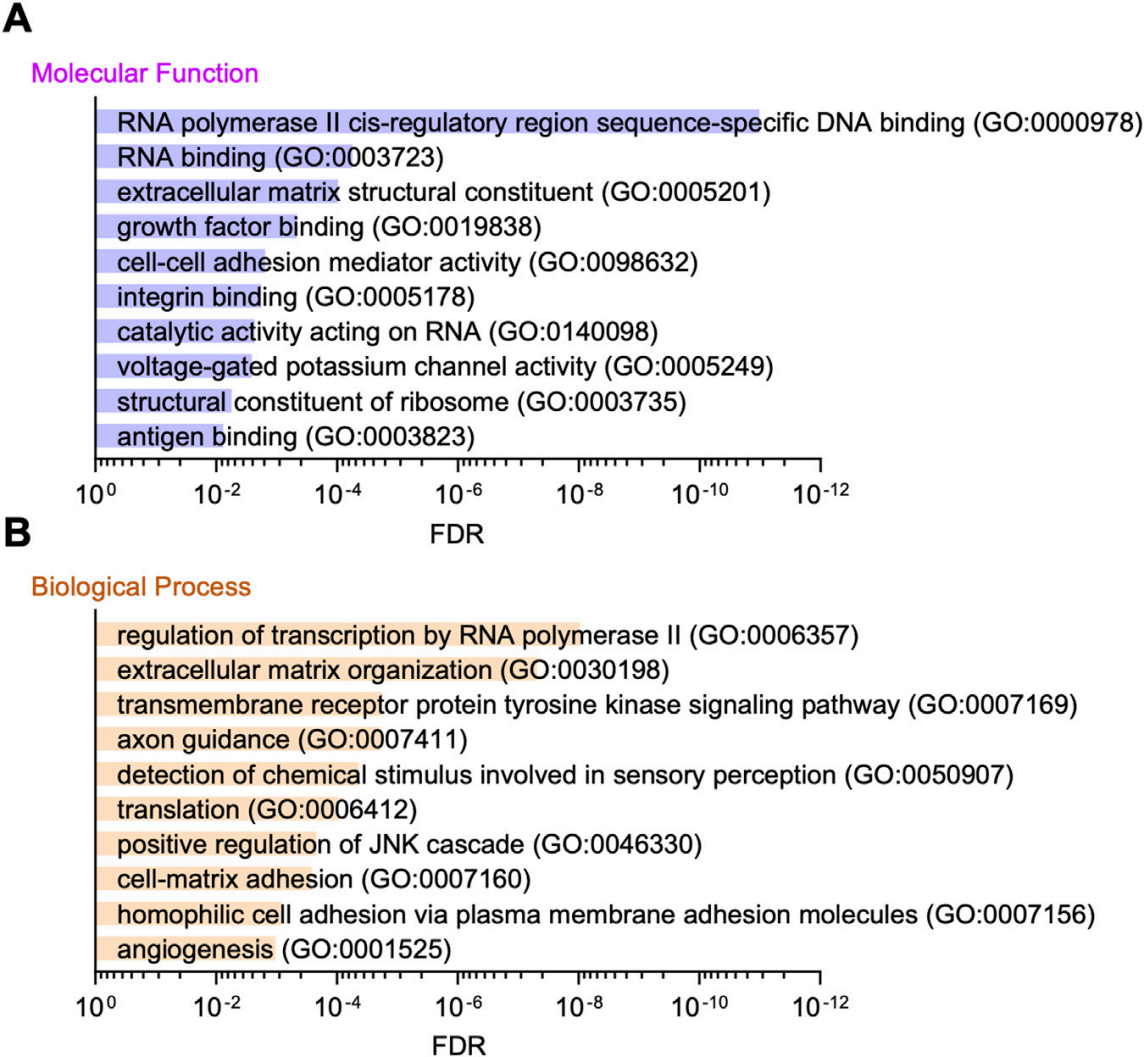
Gene ontology analysis of RNA-seq data. **(A)** Gene ontology molecular function analysis of the DEGs in RNA-seq is presented. **(B)** Gene ontology analysis for biological processes is shown.

To decipher the EGFR-bypass mechanism underlying erlotinib resistance, we performed RNA-seq analysis in Parental and ER cells. We detected a total of 30,251 genes (Supplementary Table S1). Analysis of differentially expressed genes (DEGs) with a cut-off value of adjusted p-value <0.01 and log2 fold change >8 identified 2,629 DEGs. Gene ontology molecular function (GO:MF) analysis of these DEGs showed that the top enriched gene group is RNA polymerase II cis-regulatory region sequence-specific DNA binding (Figure 3A). Consistent with this, gene ontology analysis for biological processes (GO:BP) also revealed that the top enriched group is regulation of transcription by RNA polymerase II (Figure 3B). Since RNA polymerase II mediates mRNA transcription, these data suggest that alterations in transcription may play a vital role in the EGFR-bypass mechanism for erlotinib resistance in ER cells.

**FIGURE 4:**
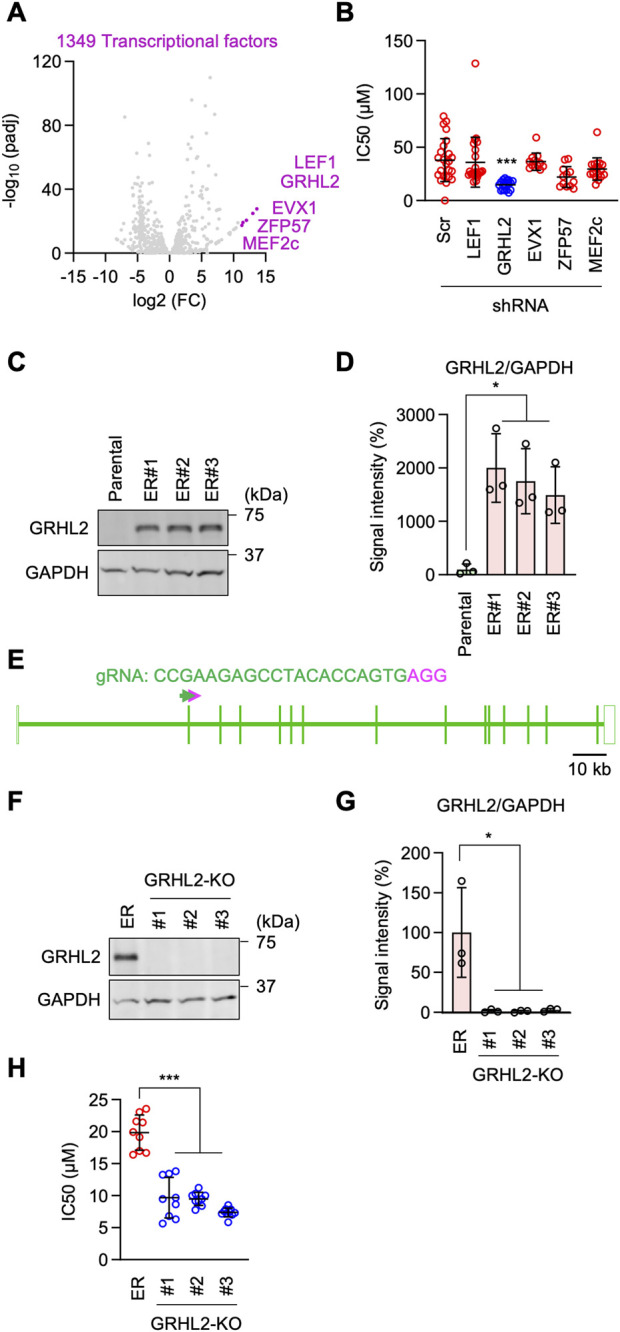
GRHL2 is critical for erlotinib resistance in ER cells. **(A)** A volcano plot showing transcription factors identified in RNA-seq analysis. The top five transcription factors are highlighted. **(B)** These five genes were knocked down in ER cells using shRNAs. IC_50_ against erlotinib was determined in each knockdown cell line. Mean ± SD (n = 24 for scramble, 24 for LEF1, 18 for GRHL2, 12 for EVX1, 12 for ZFP57, 18 for MEF2c). **(C)** Western blotting of Parental and ER cells using antibodies to GRHL2 and GAPDH. **(D)** Quantification of band intensity is shown. Mean ± SD (n = 3). **(E)** gRNA targeting exon 2 was used to knock out GRHL2 using CRISPR/Cas. **(F)** Western blotting of ER and three independent ER-GRHL2-KO cell lines using antibodies to GRHL2 and GAPDH. **(G)** Quantification of band intensity is shown. Mean ± SD (n = 3). **(H)** IC_50_ against erlotinib was determined in ER and ER-GRHL2-KO cells. Mean ± SD (n = 9 for ER, 9 for #1, 9 for #2, 10 for #3). One-way ANOVA with post hoc Tukey in **(B, D, G, H)**: *p < 0.05, ***p < 0.001.

To test the role of mRNA transcription in erlotinib resistance, we identified 1,349 transcription factors in our RNA-seq analysis and focused on the top five whose mRNA levels were highly upregulated in ER cells (Figure 4A; Supplementary Table S1). These include LEF1, GRHL2, EVX1, ZFP57, and MEF2C (Figure 4A). We individually knocked them down in ER cells using shRNAs and determined the impact on the IC_50_ of erlotinib. We found that the knockdown of GRHL2 (grainyhead-like 2), but not the other four transcription factors, significantly decreased the IC_50_ relative to a scramble control (Figure 4B). These data indicate that GRHL2 is vital for resistance to EGFR inhibition in ER cells.

GRHL2 is a member of the highly conserved grainyhead-like transcription factor family, which plays an important role in the development, function, and integrity of epithelial cells, controlling processes such as neural tube closure, epithelial barrier function, and wound healing (Mlacki et al., 2015; Frisch et al., 2017; Kotarba et al., 2020). Additionally, GRHL2 has been implicated in tumorigenesis, particularly in epithelial-mesenchymal transition (Mlacki et al., 2015; Frisch et al., 2017; Kotarba et al., 2020). However, the role of GRHL2 in drug resistance against EGFR inhibition has not been previously reported. Western blotting confirmed that GRHL2 protein abundance is dramatically upregulated in ER cells (Figures 4C, D). To further test the role of GRHL2 in ER cells, we knocked it out using the CRISPR/Cas9 genome editing system (Figure 4E; Supplementary Figure S2). The complete loss of the GRHL2 protein was confirmed by Western blotting (Figures 4F, G). Supporting the data from shRNA knockdown, the knockout of GRHL2 significantly reversed the IC_50_ for erlotinib in ER-GRHL2-KO cells (Figure 4H). Taken together, these data demonstrate that increased expression of GRHL2 mediates EGFR-bypass erlotinib resistance.

### HER3 functions in EGFR-bypass resistance downstream of GRHL2

**FIGURE 5:**
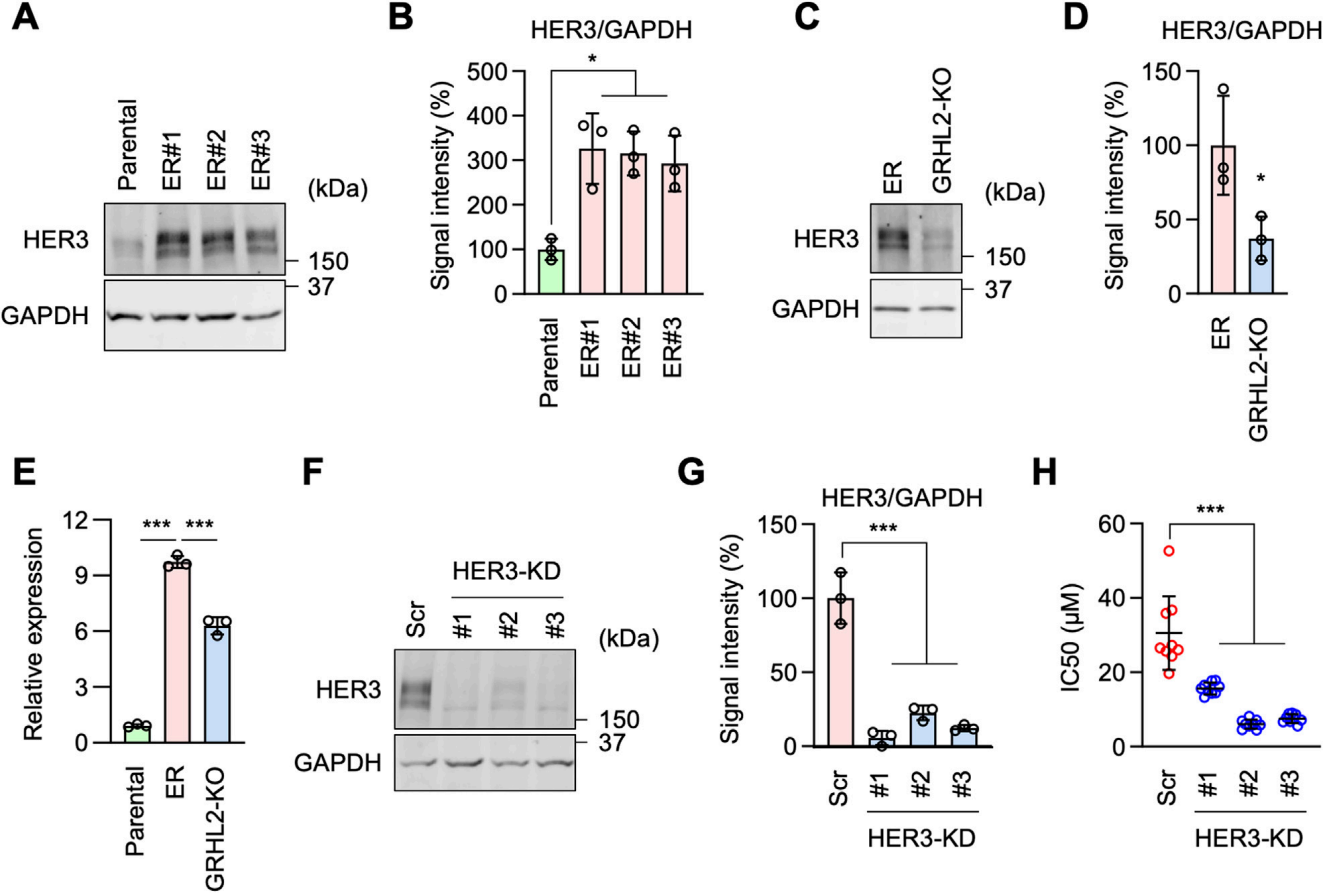
HER3 is upregulated by GRHL2 and mediates erlotinib resistance in ER cells. **(A)** Western blotting of Parental and ER cells using antibodies to HER3 and GAPDH. **(B)** Quantification of band intensity is shown. Mean ± SD (n = 3). **(C)** Western blotting of ER and ER-GRHL2-KO cells using antibodies to HER3 and GAPDH. **(D)** Quantification of band intensity is shown. Mean ± SD (n = 3). **(E)** qPCR analysis of HER3 expression in Parental, ER, and ER-GRHL2-KO cells. Quantification of HER3 transcript levels relative to GAPDH expression is shown. Mean ± SD (n = 3). **(F)** Gene knockdown of HER3 in ER cells. **(G)** Quantification of band intensity is shown. Mean ± SD (n = 3). **(H)** IC_50_ against erlotinib was determined in scramble and HER3 knockdown ER cells. Mean ± SD (n = 9). One-way ANOVA with post hoc Tukey in **(B, E, G, H)** and two-tailed Student’s t-test in **(D)**: *p < 0.05, ***p < 0.001.

To identify critical downstream components of GRHL2 in erlotinib resistance, we hypothesized that other receptor tyrosine kinases (RTKs) might be involved in this mechanism when EGFR is inhibited. Among the 53 RTKs identified in our RNA-seq data, HER3 (human epidermal growth factor receptor 3) is known to be regulated by GRHL2 (Saini et al., 2023), leading us to hypothesize that increased levels of GRHL2 promote the expression of HER3 in ER cells. Supporting this hypothesis, Western blot analysis showed that HER3 protein levels are significantly higher in ER cells compared to Parental cells (Figures 5A, B). To determine whether the increased level of HER3 depends on GRHL2, we compared HER3 protein levels in ER cells and ER-GRHL2-KO cells. Indeed, the loss of GRHL2 significantly decreased HER3 abundance (Figures 5C, D). To understand how GRHL2 controls HER3 levels, we analyzed the expression of HER3 transcripts using qPCR. We found that while HER3 transcript levels are increased in ER cells compared to Parental cells, they were only partially reduced in ER-GRHL2-KO cells (Figures 5E, F). These data suggest that GRHL2 regulates HER3 protein levels through transcription as well as additional mechanisms, such as translation or protein stability. Furthermore, to assess the functional contribution of HER3 to drug resistance, we knocked down HER3 using shRNAs in ER cells (Figure 5G). We found that HER3 knockdown significantly decreased the IC_50_ of erlotinib (Figure 5H). These data suggest that EGFR-bypass resistance to erlotinib is driven by GRHL2-dependent upregulation of HER3.

### E-cadherin is critical for EGFR-bypass drug resistance independently of GRHL2

**FIGURE 6:**
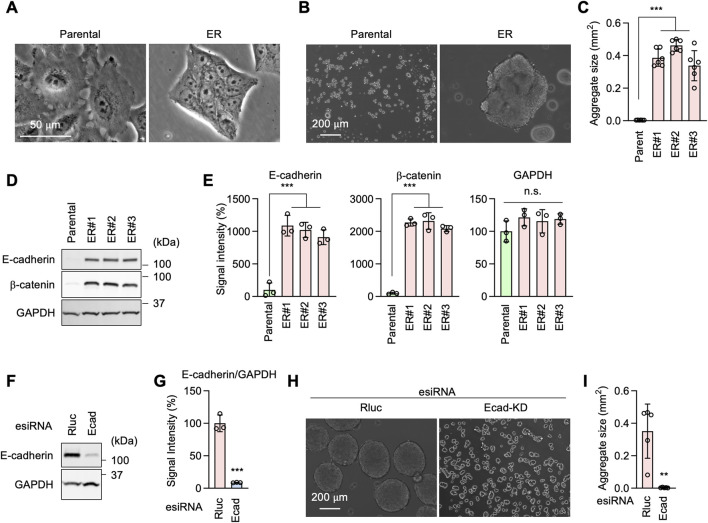
E-cadherin produces erlotinib resistance in ER cells. **(A)** Phase contrast microscopy of Parental and ER cells. **(B)** Parental and ER cells were incubated in non-adhesive cell culture plates for 24 h with gentle rotation. **(C)** The size of cell aggregates was quantified. Mean ± SD (n = 6). **(D)** Western blotting of Parental and ER cells using antibodies to E-cadherin, β-catenin, and GAPDH. **(E)** Quantification of band intensity is shown. Mean ± SD (n = 3). **(F)** Gene knockdown of E-cadherin in ER cells. **(G)** Quantification of band intensity is shown. Mean ± SD (n = 3). **(H)** Renilla luciferase (Rluc) and Ecad knockdown ER cells were analyzed in the cell-cell adhesion assay described in **(B)**. **(I)** The size of cell aggregates was quantified. Mean ± SD (n = 6). One-way ANOVA with *post hoc* Tukey in **(C, E)** and two-tailed Student’s t-test in **(G, I)**: *p < 0.05, **p < 0.01, ***p < 0.001.

During cell culture, we observed ER cells adhering to each other more than Parental cells (Figure 6A). This observation suggested that ER cells may have increased cell-cell adhesion. To test this hypothesis, we performed a cell-cell adhesion assay by incubating dissociated cells under continuous gentle shaking for 24 h, followed by measuring the size of the cell aggregates as an indicator of cell-cell adhesion. Results showed that while Parental cells remained largely dissociated, ER cells were associated with each other and formed large aggregates (Figures 6B, C). These data suggest that ER cells have an increased ability for cell-cell adhesion. Our findings are consistent with the RNA-seq data showing enrichment of cell-cell adhesion mediator activity (GO:MF) (Figure 3A) and homophilic cell adhesion via plasma membrane adhesion molecules (GO:BP) (Figure 3B).

**FIGURE 7:**
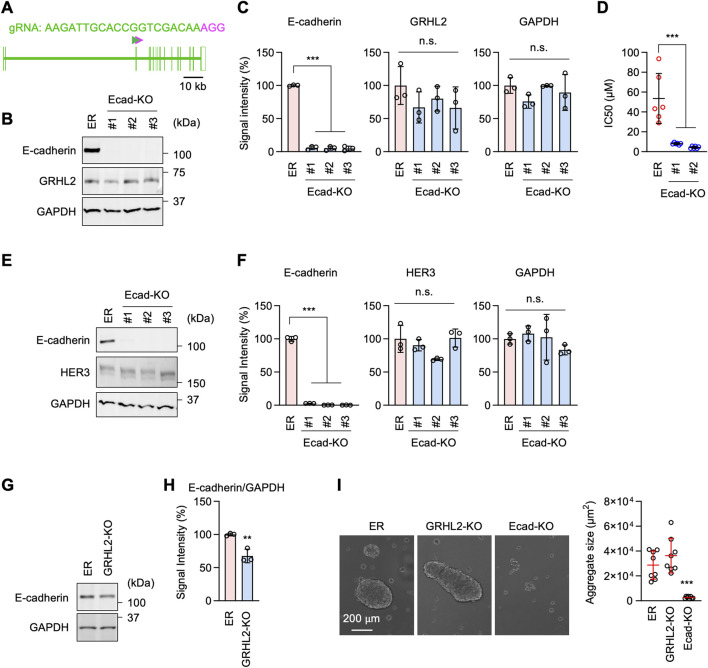
E-cadherin-mediated erlotinib resistance is independent of GRHL2 and HER3. **(A)** gRNA targeting exon 3 was used to knock out E-cadherin using CRISPR/Cas. **(B)** Western blotting of ER and three independent ER-Ecad-KO cell lines using E-cadherin, GRHL2, and GAPDH antibodies. **(C)** Quantification of band intensity is shown. Mean ± SD (n = 3). **(D)** IC_50_ against erlotinib was determined in ER and ER-Ecad-KO cells. Mean ± SD (n = 6). **(E)** Western blotting of ER and three independent ER-Ecad-KO cell lines using E-cadherin, HER3, and GAPDH antibodies. **(F)** Quantification of band intensity is shown. Mean ± SD (n = 3). **(G)** Western blotting of ER and ER-GRHL2-KO cells using antibodies to E-cadherin and GAPDH. **(H)** Quantification of band intensity is shown. Mean ± SD (n = 3). **(I)** The cell-cell adhesion assay was performed using ER, ER-GRHL2-KO, and ER-Ecad-KO cells. The size of cell aggregates was quantified. Mean ± SD (n = 5). One-way ANOVA with post hoc Tukey in **(C, D, F, I)** and Student’s t-test in **(H)**: *p < 0.05, **p < 0.01, ***p < 0.001.

Western blotting showed that the cell adhesion molecule E-cadherin and its functional partner, β-catenin, are considerably upregulated in ER cells (Figures 6D, E). To test the role of E-cadherin in increased cell adhesion in ER cells, we knocked down E-cadherin using esiRNAs (Figures 6F, G). E-cadherin knockdown greatly decreased cell-cell adhesion in our cell aggregation assay (Figures 6H, I). To assess the role of E-cadherin in drug resistance, we knocked E-cadherin using CRISPR/Cas9 in ER cells (Figures 7A–C). The loss of E-cadherin was confirmed by Western blotting (Figure 7B). Strikingly, ER-Ecad-KO cells exhibited a significantly reduced IC_50_ for erlotinib (Figure 7D). These data demonstrate that E-cadherin is critical for EGFR-bypass drug resistance.

To investigate the relationship between the roles of E-cadherin and GRHL2-HER3, we probed GRHL2 and HER3 levels in ER-Ecad-KO cells using Western blotting. The results showed that the loss of E-cadherin does not affect the protein levels of GRHL2 (Figures 7B, C) or HER3 (Figures 7E, F) in ER-Ecad-KO cells. Therefore, the change in IC_50_ in ER-Ecad-KO cells is not due to the loss of GRHL2 or HER3.

To determine whether GRHL2 regulates E-cadherin expression, we performed Western blotting on ER-GRHL2-KO cells and found that E-cadherin levels were slightly decreased in ER-GRHL2-KO cells compared to ER cells (Figures 7G, H). Consistent with the largely remaining expression of E-cadherin, ER-GRHL2-KO cells maintained enhanced cell-cell adhesion and formed large cell aggregates in the cell adhesion assay (Figure 7I). In contrast, ER-Ecad-KO cells exhibited a significant decrease in cell-cell adhesion, as expected (Figure 7I). These data indicate that the increased levels of E-cadherin are primarily independent of GRHL2 and that E-cadherin and GRHL2 mediate EGFR-bypass drug resistance through distinct pathways.

### Intracellular signaling pathways involved in EGFR-bypass drug resistance

**FIGURE 8:**
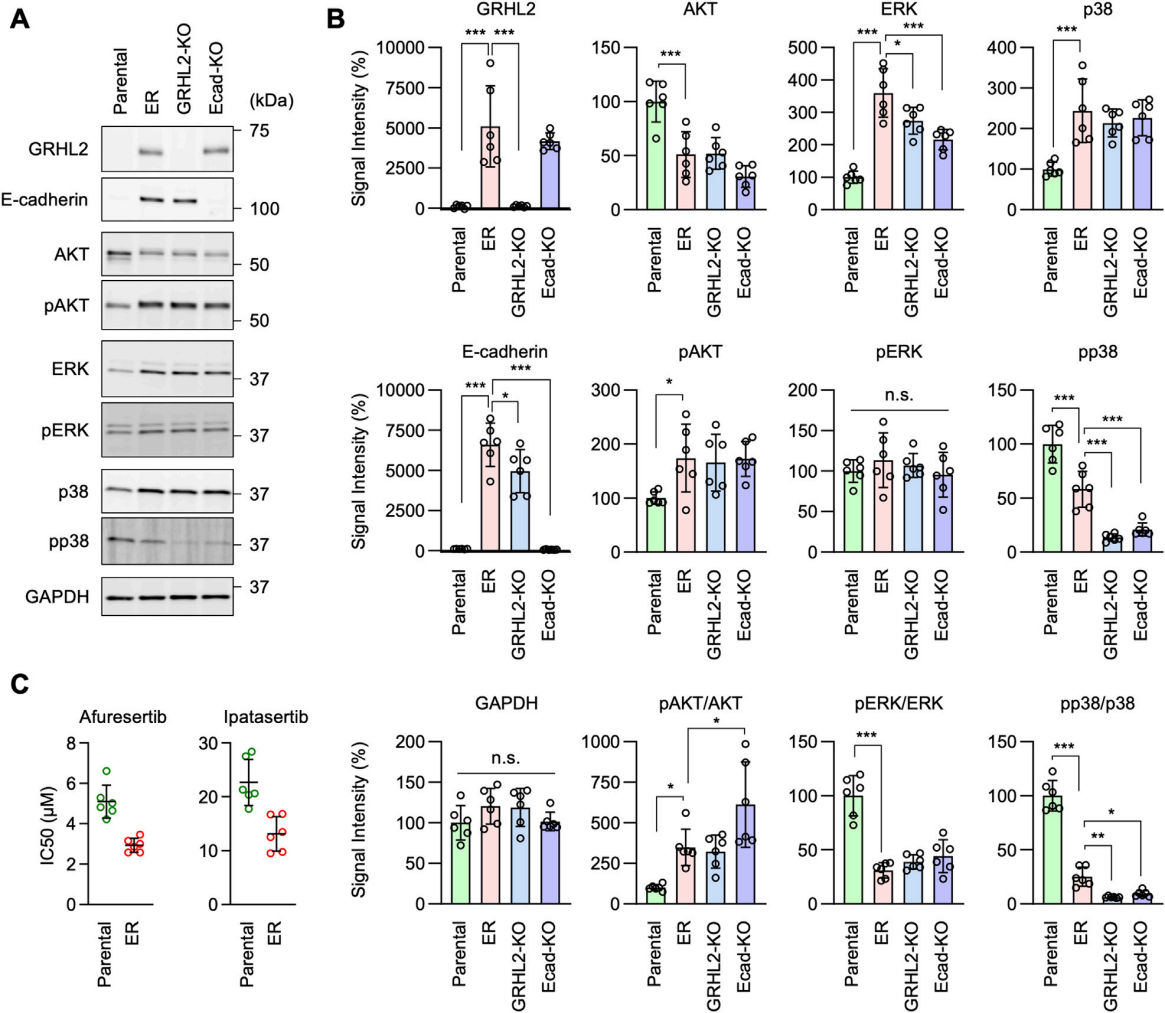
AKT phosphorylation is important for erlotinib resistance in ER cells. **(A)** Western blotting of Parental, ER, ER-GRHL2-KO, and ER-Ecad-KO cells using the indicated antibodies. **(B)** Quantification of band intensity is shown. Mean ± SD (n = 5). **(C)** IC_50_ against afuresertib and ipatasertib was determined in Parental and ER cells. Mean ± SD (n = 6). One-way ANOVA with post hoc Tukey in **(B)** and Student’s t-test in **(C)**: *p < 0.05, **p < 0.01, ***p < 0.001.

To further investigate the mechanisms underlying EGFR-bypass drug resistance in ER cells, we analyzed intracellular signal transduction pathways involving the phosphorylation of AKT, ERK1/2, and p38 using Western blotting, since these proteins can function downstream of HER3. First, we found that the level of AKT S473 phosphorylation was elevated in ER cells (Figures 8A,B, pAKT). Since the protein level of AKT was decreased in ER cells (Figures 8A, B, AKT), the proportion of phosphorylated AKT was much higher than in Parental cells (Figures 8A, B, pAKT/AKT). Second, while ERK1/2 phosphorylation levels were not changed in ER cells (Figures 8A,B, pERK), the protein levels of ERK1/2 were increased (Figures 8A, B, ERK), leading to a reduced proportion of phosphorylated ERK1/2 in these cells (Figures 8A, B, pERK/ERK). Third, p38 phosphorylation levels were decreased in ER cells (Figures 8A, B, pp38), while their protein levels were increased (Figures 8A, B, p38). These data suggest that the AKT pathway plays a crucial role in conferring drug resistance to erlotinib in ER cells. Supporting the critical role of AKT in ER cells, we found that ER cells were more sensitive to AKT inhibitors compared to Parental cells: the IC_50_ of two different AKT inhibitors (afuresertib and ipatasertib) was lower in ER cells (Figure 8C).

**FIGURE 9:**
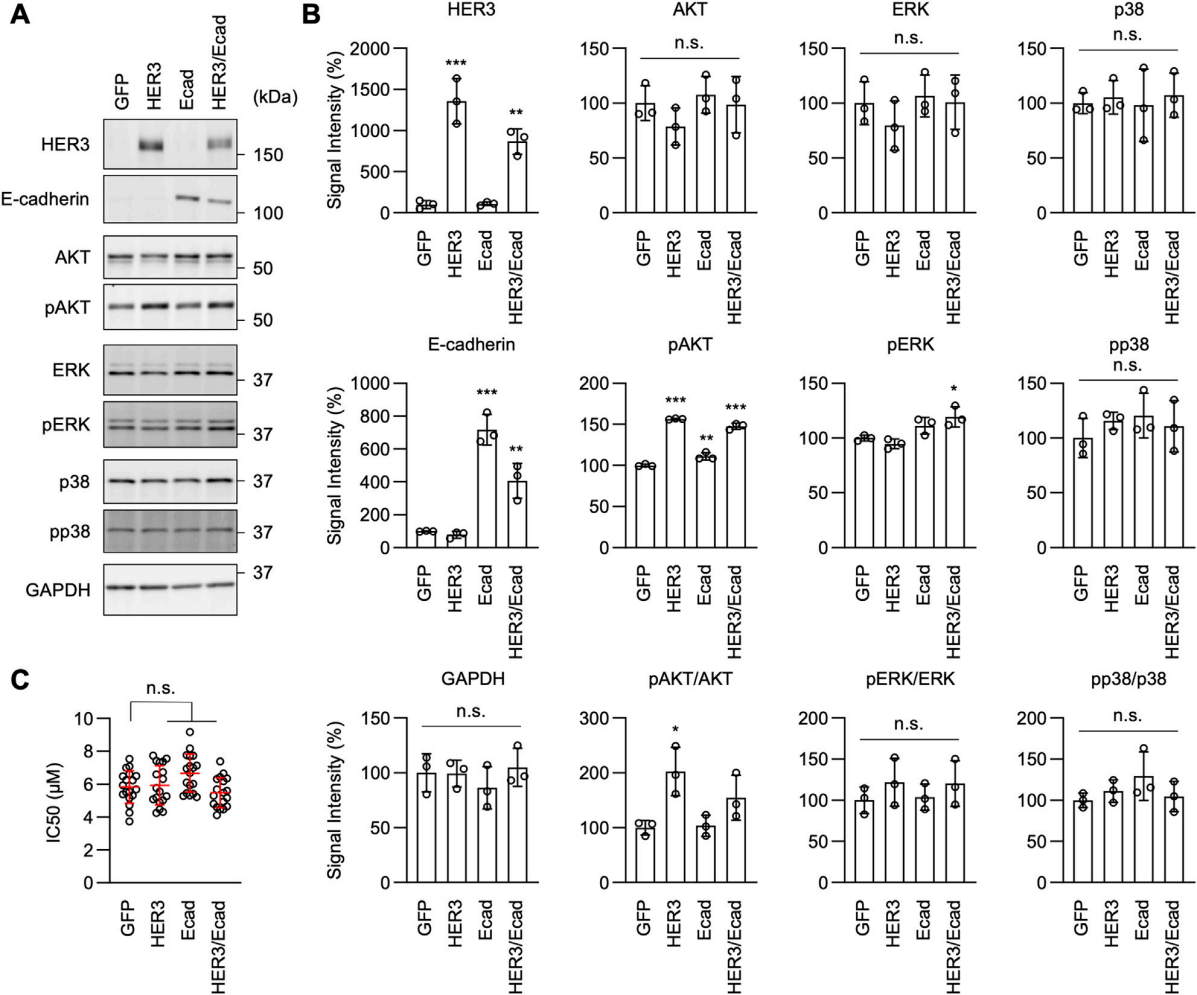
AKT phosphorylation is important for erlotinib resistance in ER cells. **(A)** Western blotting of Parental cells overexpressing GFP, HER3, E-cadherin, and both HER3 and E-cadherin using the indicated antibodies. **(B)** Quantification of band intensity is shown. Mean ± SD (n = 3). **(C)** IC_50_ against erlotinib was determined in the same set of cells. Mean ± SD (n = 18). One-way ANOVA with post hoc Tukey in **(B, C)**: *p < 0.05, **p < 0.01, ***p < 0.001.

Interestingly, we found that AKT phosphorylation levels remained elevated in ER-GRHL2-KO and ER-Ecad-KO cells (Figures 8A, B, pAKT), while p38 phosphorylation decreased further in these knockout cells (Figures 8A, B, pp38). These data suggest that drug resistance involves increased AKT phosphorylation and that the loss of drug resistance in ER-GRHL2-KO and ER-Ecad-KO cells is accompanied by decreased p38 phosphorylation. If this is the case, increased AKT phosphorylation alone may not be sufficient for drug resistance. To test this hypothesis, we overexpressed HER3 and E-cadherin individually and in combination in Parental cells using lentiviruses and measured the IC_50_ for erlotinib. The results showed that while HER3 overexpression or its combination with E-cadherin overexpression enhanced AKT phosphorylation (Figures 9A, B, pAKT), they did not induce drug resistance (Figure 9C). Therefore, although HER3 and E-cadherin are necessary for drug resistance, their overexpression alone is not sufficient, likely requiring the involvement of other components.

## Discussion

The development of erlotinib resistance presents a significant challenge in treating NSCLC (Herbst et al., 2018; Leiter et al., 2023; Liu et al., 2023). Our study established an EGFR-independent erlotinib-resistant phenotype by continuously exposing A549 cells to sublethal doses of erlotinib over 6 months. The resultant ER cells demonstrated a dramatic increase in IC_50_ for erlotinib, with resistance levels up to 10 times greater than those of Parental cells. Notably, these ER cells showed enhanced tumorigenic potential both in 3D cultures and *in vivo*, forming significantly larger tumors than Parental cells. This indicates that ER cells have developed a robust mechanism to bypass EGFR inhibition and promote tumor growth, which is crucial for understanding resistance mechanisms.

Unexpectedly, our investigation into this resistance’s mechanisms revealed that ER cells did not rely on traditional pathways associated with increased EGFR expression or common activating EGFR mutations (Rosell et al., 2009; Yu et al., 2014; Sattler et al., 2023; Tomuleasa et al., 2024). Instead, EGFR protein levels were found to be decreased in ER cells. These findings suggest that the resistance mechanism in these ER cells is independent of direct alterations in the EGFR pathway. This observation aligns with emerging evidence that some resistant cancers can activate alternative pathways or utilize bypass signaling mechanisms to maintain proliferation despite EGFR inhibition (Chong and Janne, 2013; Sever and Brugge, 2015; Rotow and Bivona, 2017; Leonetti et al., 2019; Passaro et al., 2021; Cooper et al., 2022; Tomuleasa et al., 2024).

Our RNA-seq analysis provided further insight into the molecular underpinnings of this EGFR-bypass resistance. We identified GRHL2, a transcription factor, as a critical player in this process. GRHL2 was significantly upregulated in ER cells, and its knockdown and knockout markedly reduced the IC_50_ for erlotinib, highlighting its essential role in maintaining resistance. This is further supported by the increased expression of HER3, a receptor tyrosine kinase known to be regulated by GRHL2, in ER cells. Knockdown of HER3 similarly reduced erlotinib resistance, suggesting that GRHL2-mediated upregulation of HER3 contributes to the bypass mechanism. These findings implicate the GRHL2-HER3 axis as a potential therapeutic target in overcoming erlotinib resistance. Since HER3 has minimal or no intrinsic tyrosine kinase activity (Tomuleasa et al., 2024), it likely mediates drug resistance through interactions with other tyrosine kinases.

In addition to the GRHL2-HER3 pathway, we identified E-cadherin as another key factor in the EGFR-bypass resistance mechanism. ER cells exhibited increased cell-cell adhesion, associated with the upregulation of E-cadherin and its partner, β-catenin. The knockout of E-cadherin reduced cell-cell adhesion and significantly decreased the IC_50_ for erlotinib. Notably, the finding that E-cadherin expression is largely independent of GRHL2 suggests that multiple parallel pathways converge to sustain the EGFR-bypass resistance in ER cells. This notion is further supported by the fact that GRHL2 is not involved in increased cell-cell adhesion in ER cells. This dual mechanism of resistance underscores the complexity of targeting EGFR in NSCLC. It suggests that combination therapies targeting both GRHL2-HER3- and E-cadherin-mediated pathways might be necessary to overcome resistance in patients effectively.

We identified elevated AKT S473 phosphorylation as a potential vital mechanism in drug resistance in ER cells. Consistently, ER cells rely on AKT signaling and are more sensitive to AKT inhibitors compared to Parental cells. In contrast, ERK1/2 phosphorylation remained unchanged, and p38 phosphorylation decreased. However, increased AKT phosphorylation alone is insufficient to induce drug resistance, as overexpressing HER3 did not confer resistance in Parental cells, indicating that additional factors are necessary for the resistant phenotype.

Although the mechanisms underlying the upregulation of GRHL2 and E-cadherin in ER cells remain unknown, epigenetic changes may influence their expression (Glasspool et al., 2006; Laisne et al., 2024). For example, hypomethylation of GRHL2 and E-cadherin promoters could increase their expression, enhancing cell adhesion and resistance. Additionally, changes like histone acetylation or methylation can make the GRHL2 and E-cadherin genes more accessible for transcription, thereby elevating their expression in response to drug resistance. Furthermore, the downregulation of miRNAs that typically suppress GRHL2 and E-cadherin, or the upregulation of lncRNAs that enhance their stability, could boost these genes’ expression in resistant cells. These potential epigenetic mechanisms offer a potential explanation for the atypical expression of GRHL2 and E-cadherin and suggest opportunities for targeting resistance at the epigenetic level.

GRHL2 and E-cadherin are linked to epithelial cell characteristics, and their decreased levels are often associated with epithelial-mesenchymal transition (EMT) (Dongre and Weinberg, 2019; Yang et al., 2020; Youssef and Nieto, 2024). EMT is a dynamic process that promotes the reorganization of cellular functions and behaviors during embryonic development (Dongre and Weinberg, 2019; Yang et al., 2020; Youssef and Nieto, 2024). During EMT, cellular polarity is lost, cell-cell adhesion is decreased, and cell motility is increased. Importantly, EMT also occurs during cancer development and drug resistance (Dongre and Weinberg, 2019). Therefore, the increased levels of GRHL2 and E-cadherin, along with the enhanced cell-cell adhesion observed in erlotinib-resistant ER cells, are contrary to what is typically expected and quite surprising.

Cancer cells can undergo a partial or hybrid EMT, retaining epithelial features (like E-cadherin expression) while adopting mesenchymal traits (Jolly et al., 2022). This hybrid state may allow cells to survive drug pressure without fully losing epithelial characteristics. Some studies suggest that while low E-cadherin is generally linked to invasiveness, maintaining it can confer drug resistance in ovarian cancer (Xu et al., 2014). Additionally, E-cadherin can interact with pathways like PI3K/AKT, supporting survival and resistance rather than promoting invasion (Mendonsa et al., 2018). In future studies, it will be important to investigate how some epithelial characteristics may contribute to drug resistance and potentially EGFR-independent activation of signal transduction in NSCLC cells.

Our findings could contribute to the development of future therapeutic strategies to combat erlotinib resistance. First, the identification of GRHL2 as a transcription factor driving erlotinib resistance, along with the subsequent upregulation of HER3, provides a basis for designing therapies that inhibit both GRHL2 and HER3, given that HER3 inhibitors such as HER3-DXd are currently under investigation for NSCLC (Yu et al., 2023). Second, E-cadherin independently contributes to resistance mechanisms. Therapeutics designed to disrupt or modulate E-cadherin-mediated adhesion may help mitigate this resistance pathway. Third, our findings imply that single-agent therapies may not be sufficient to overcome erlotinib resistance due to the presence of parallel pathways sustaining the resistance phenotype. Therefore, a combinatory approach targeting both GRHL2-HER3 and E-cadherin-mediated cell adhesion may more effectively dismantle the multifaceted resistance in these cancer cells. Fourth, GRHL2, HER3, and E-cadherin levels could serve as biomarkers to monitor treatment efficacy or predict the onset of resistance in patients undergoing erlotinib treatment. Tailoring treatment regimens based on the expression levels of these markers may personalize therapy, optimizing response rates and potentially delaying or preventing resistance.

Translating findings from cell line studies to clinical practice is often challenging due to differences in tumor microenvironments (TMEs) and patient-specific factors (Pouyssegur et al., 2006; Quail and Joyce, 2013; Coban et al., 2021; Gu et al., 2024; Mai et al., 2024; Wu et al., 2024). The tumor microenvironment may modulate GRHL2 and E-cadherin expression in drug-resistant cells. First, TME-derived factors like TGF-β and EGF can either suppress or enhance GRHL2 and E-cadherin expression (Gu et al., 2024). TGF-β, for instance, often downregulates E-cadherin, promoting invasion, while certain TME signals may upregulate both proteins, enhancing cell-cell adhesion and supporting collective cell survival under drug pressure. Second, hypoxia in the TME may reduce E-cadherin levels via HIF signaling, fostering an invasive phenotype (Pouyssegur et al., 2006). However, under metabolic stress, cells might rely more on E-cadherin-mediated adhesion for survival. GRHL2 can also respond to such stress, promoting epithelial traits that contribute to resistance. Third, stromal cancer-associated fibroblasts (CAFs) and immune cells can influence GRHL2 and E-cadherin through secreted factors (Coban et al., 2021). CAFs and immune cytokines support adhesion and survival signaling, reinforcing drug-resistant phenotypes in tumor cells. Fourth, ECM components and stiffness within the TME may upregulate E-cadherin and GRHL2 (Mai et al., 2024). This enhanced adhesion and structural support contribute to cell survival and resistance under therapeutic stress. In future studies, it is critical to address these key aspects using patient-derived xenografts and organoid models, which better preserve the TME’s influence on cancer cell behavior.

There are several key questions that remain to be addressed. For instance, we primarily used A549 cells in both 2D and 3D *in vitro* culture systems. While A549 cells are a widely used model for studying NSCLC and erlotinib resistance, they represent a single cell line with specific genetic and phenotypic traits that may not fully reflect the heterogeneity of NSCLC in patients. Additionally, erlotinib may have off-target effects that could influence cell behavior independently of EGFR inhibition, potentially impacting our observations on resistance mechanisms. Such effects might lead to unintended activation or repression of pathways unrelated to EGFR, which could affect GRHL2, HER3, or E-cadherin expression and contribute to resistance in ways not solely dependent on EGFR signaling. To address these limitations, we plan to use multiple NSCLC cell lines and patient-derived models in future studies, as well as examine erlotinib’s specificity using EGFR mutations that are not inhibited by erlotinib. Expanding model systems and assessing off-target effects will help clarify the broader applicability of these resistance mechanisms in a clinical context.

## Data Availability

The RNA-seq data generated in this study have been deposited in the Gene Expression Omnibus database (Accession code PRJNA1156760). All other data are provided in the main text or the Supplementary Materials.
