# Supplementary material for: GRHL2-HER3 and E-cadherin mediate EGFR-bypass drug resistance in lung cancer cells
Source: Front Cell Dev Biol. 2025 Jan 17;12:1511190. doi: 10.3389/fcell.2024.1511190 (PMC11782226; doi:10.3389/fcell.2024.1511190)
Supplement: Supplementary file 1 [file DataSheet1.pdf]

Supplementary Material

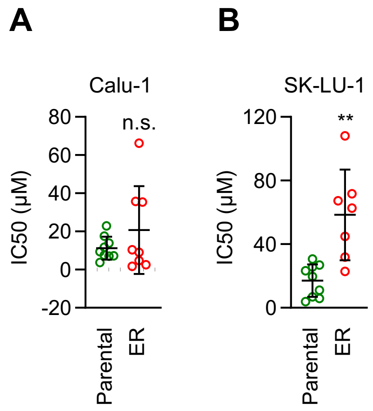

Supplementary Figure 1. (A and B) Calu-1 cells (A) and SK-LU-1 cells (B) were treated with 10–30 µM erlotinib for six months. The IC_{50} values against erlotinib were measured in parental and erlotinib-treated cells (ER cells). Mean ± SD (n = 7–9). Student's t-test: **p < 0.01.

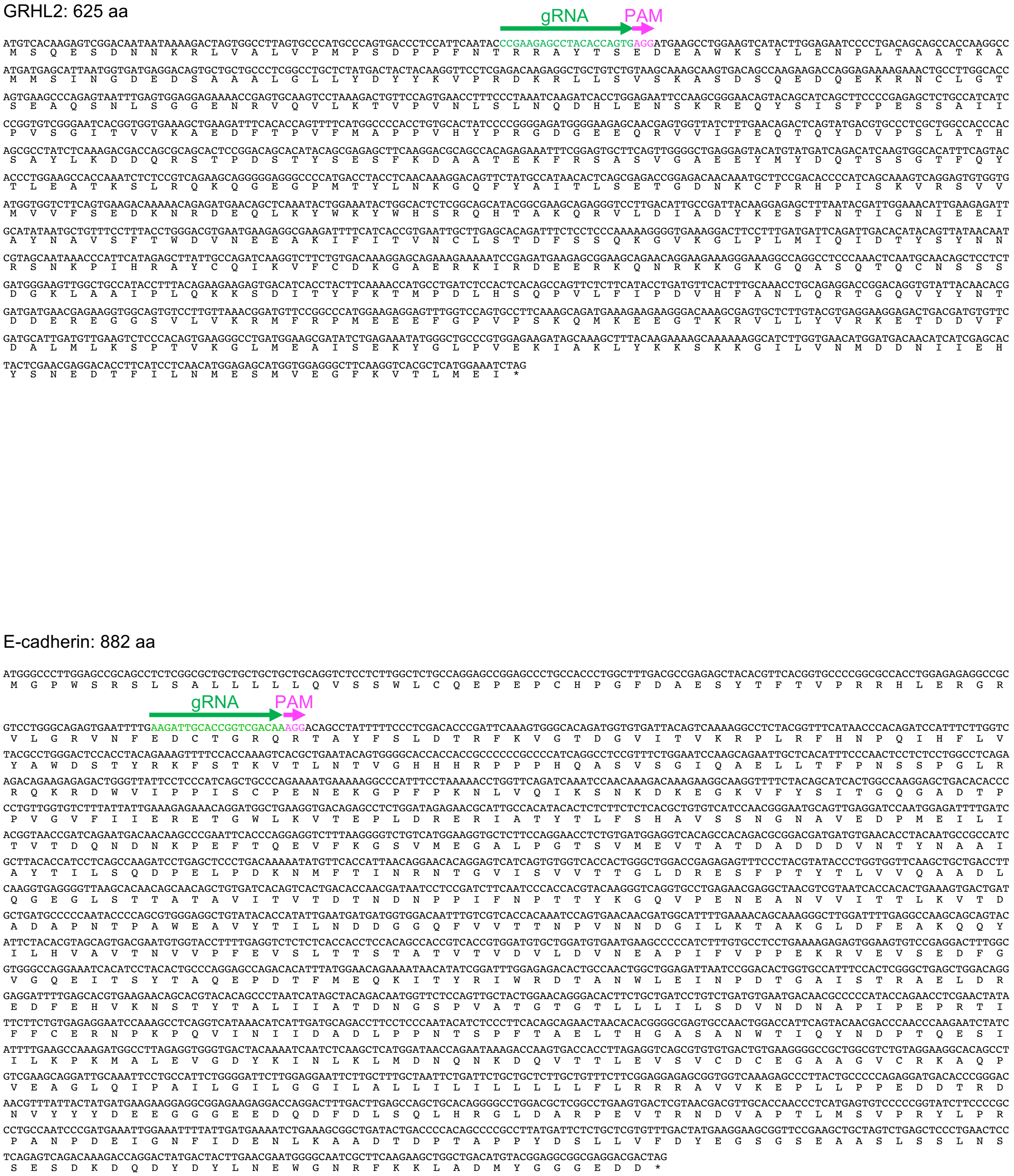

Supplementary Figure 2. Knockout of GRHL2 and E-cadherin using CRISPR. GRHL2 and E-cadherin were knocked out in ER cells using the indicated gRNA sequence.
